# The Landscape of the DNA Transposons in the Genome of the Horezu_LaPeri Strain of *Drosophila melanogaster*

**DOI:** 10.3390/insects14060494

**Published:** 2023-05-25

**Authors:** Alexandru Marian Bologa, Ileana Stoica, Nicoleta Denisa Constantin, Alexandru Al. Ecovoiu

**Affiliations:** Department of Genetics, Faculty of Biology, University of Bucharest, 060101 Bucharest, Romania; alexandru.bologa@drd.unibuc.ro (A.M.B.); ileana.stoica@bio.unibuc.ro (I.S.); alexandru.ecovoiu@bio.unibuc.ro (A.A.E.)

**Keywords:** transposable elements, DNA natural transposons, *Drosophila melanogaster*, P-element, heterochromatin, bioinformatics, transposon annotation

## Abstract

**Simple Summary:**

Transposons are mobile genetic elements that can mobilize to other locations in the host genomes. Generally, the mapping of natural transposons is a meticulous endeavor, since the repetitive nature of these elements impedes on the accurate localization of the insertions. In this study, we outlined the landscape of the DNA transposons of a natural population of *Drosophila melanogaster* from Romania. Harnessing a set of bioinformatics tools designed for transposon mapping, we identified multiple insertions affecting genes with a potential adaptive role. One focus of our work was a detailed analysis of a recently invading transposon, known as the P-element. Another target was to map the DNA natural transposons located in various heterochromatic regions, known as preferential targets for insertions. Our research contributes to a better understanding of the dynamics of DNA transposons and of their roles in the structure and evolution of the *D. melanogaster* genome.

**Abstract:**

Natural transposons (NTs) represent mobile DNA sequences found in both prokaryotic and eukaryotic genomes. *Drosophila melanogaster* (the fruit fly) is a eukaryotic model organism with NTs standing for about 20% of its genome and has contributed significantly to the understanding of various aspects of transposon biology. Our study describes an accurate approach designed to map class II transposons (DNA transposons) in the genome of the Horezu_LaPeri fruit fly strain, consecutive to Oxford Nanopore Technology sequencing. A whole genome bioinformatics analysis was conducted using Genome ARTIST_v2, LoRTE and RepeatMasker tools to identify DNA transposons insertions. Then, a gene ontology enrichment analysis was performed in order to evaluate the potential adaptive role of some DNA transposons insertions. Herein, we describe DNA transposon insertions specific for the Horezu_LaPeri genome and a predictive functional analysis of some insertional alleles. The PCR validation of P-element insertions specific for this fruit fly strain, along with a putative consensus sequence for the KP element, is also reported. Overall, the genome of the Horezu_LaPeri strain contains several insertions of DNA transposons associated with genes known to be involved in adaptive processes. For some of these genes, insertional alleles obtained via mobilization of the artificial transposons were previously reported. This is a very alluring aspect, as it suggests that insertional mutagenesis experiments conducting adaptive predictions for laboratory strains may be confirmed by mirroring insertions which are expected to be found at least in some natural fruit fly strains.

## 1. Introduction

Natural transposons (NTs) are relatively short repetitive DNA sequences found in both prokaryotic and eukaryotic genomes, influencing the size, structure and functionality of genomes [1]. Depending on the method of transposition, NTs have been classified into two classes: class I elements (retrotransposons) that involve an intermediate RNA molecule in the transposition process, and class II elements (DNA transposons), whose transposition involves DNA excision and repair [2]. 

The total NT content of *Drosophila melanogaster* was estimated to be ~20% of its genome. The most representative types are long terminal repeat (LTR-type) retrotransposons and long interspersed nuclear elements (LINEs). Regarding class II transposons in *D*. *melanogaster*, the most prominent are the terminal inverted-repeat elements (TIRs) and Helitrons [3]. One of the best studied DNA transposons in eukaryotic genomes is the P-element. Following horizontal gene transfer from the genome of *Drosophila willistoni* to the genome of *D*. *melanogaster* [4], P elements spread via vertical gene transfer and currently form a heterogeneous family that includes both the complete ancestral sequence and a collection of the P-element’s derivative sequences. In natural populations of *D*. *melanogaster*, both autonomous P elements (the complete length is 2907 nucleotides) and smaller, non-autonomous P elements harboring internal deletions may be present [5]. One of the most common non-autonomous P elements is the KP element, which contains a deletion reported at similar genomic coordinates: 808–2560 [6,7], 807–2561 [8] or 809–2561 [9]. While the integral P-element is capable of encoding the transposase enzyme able to recognize the transposon’s inverted repeat ends (31 bp) to initiate transposition, the protein encoded by the KP element is an important repressor of P-element transposition [6,9,10]. Similar to other class II transposons, the P-element produces a target site duplication (TSD) of a length of eight nucleotides that flanks the two TIRs of the transposon. Over the years, efforts have been made in order to identify the sequence motifs for which the P-element has an insertion affinity. An eight-nucleotide consensus sequence (GGCCAGAC) was originally proposed in 1983 [11], and more recently, a 14-nucleotide palindromic consensus sequence (ATRGTCCGGACWAT) was evidenced as the most probable target for the P-element [12].

Mapping and annotation of NTs are two of the most difficult analyses in bioinformatics and computational genomics. Currently, the analysis of sequencing data containing transposons from different populations has benefited from the development of new sequencing strategies, as well as from the development of applications that detect repetitive sequence patterns [13]. Before the development of long-read sequencing technologies such as Oxford Nanopore Technologies (ONT) and Pacific Biosciences, NTs were detected via short-read sequencing and investigation of the junction region between the transposon and the genomic flanking sequence. Because of the relative ambiguity of the short read’s alignments (multi-mapping), transposons are difficult to map, and quite often the programs do not distinguish between different copies of the same transposon [14,15,16]. Long reads can span both the total length of a transposon and of its flanking regions, allowing for a more precise mapping of the transposons, an aspect which alleviates the problem of ambiguous alignments [17]. As a drawback, since the long-read sequencing technologies are relatively recent [18,19], the number of bioinformatics programs exploiting long reads for NT mapping and analysis is limited.

Herein we report data on the content of DNA transposons identified in the genome of the natural population of *D*. *melanogaster* Horezu_LaPeri, with a focus on the P-element. The evaluation of transposon content is based on the analysis of the long reads obtained following the sequencing of the genome of interest (using LoRTE [20]), as well as on the analysis of the contigs obtained following Canu assembly (investigated with Genome ARTIST_v2 [21] and RepeatMasker [22] bioinformatics tools). We report the genomic location at nucleotide and chromosomal levels for each DNA transposon mapped using GA_v2, regardless of if it is specific for Horezu_LaPeri or conserved relative to the reference genome (r6.48). In order to infer functional similarities predictions for a selection of genes affected by NTs insertions, we performed a gene ontology (GO) enrichment analysis using the enrichment software FlyEnrichr [23]. Additionally, we computed a putative consensus sequence for KP elements, and we validated via polymerase chain reaction (PCR) some of the P-element/KP insertions.

## 2. Materials and Methods

### 2.1. Nanopore Sequencing and Genome Assembly

Sequencing data used in this study were obtained from a Romanian local natural strain of *D. melanogaster*, named Horezu_LaPeri and collected from the location Romanii de Sus, Horezu, Vâlcea County, Romania, in August 2018. Nanopore sequencing of the Horezu_LaPeri genome was performed with the MinION device from ONT and was previously described in detail elsewhere [24], along with the qualitative parameters of four alternative assemblies generated with Canu v2.1.1 [25] and Flye v2.8.3 [26] applications. In our hands, the most reliable assembly for transposon analysis was Canu-Data set I, obtained using unfiltered long reads. This Whole-Genome Shotgun project has been deposited at DDBJ/ENA/GenBank under the accession key JANZWZ000000000. The version described in this paper is version JANZWZ020000000.

### 2.2. Computational Environment

Bioinformatics analysis was mainly performed on a device equipped with 64 Gb DDR4 RAM, an AMD Ryzen 7 4800H CPU processor, 1 Tb SSD and Linux Mint 21.1 Cinnamon operating system. 

### 2.3. Identification and Mapping of DNA NT Insertions

FASTA files containing the reference genome of *D*. *melanogaster* (r6.48) and genes/NTs annotations files were downloaded from FlyBase [27] and loaded in the databases of GA_v2 (https://genomeartist.ro/, accessed on 7 December 2022). Unmapped scaffolds that could not be associated with any *D. melanogaster* reference chromosomes were eliminated. *Drosophila* NTs canonical sequences were downloaded from GitHub repository bergmanlab/drosophila-transposons (https://github.com/bergmanlab/drosophila-transposons, accessed on 7 December 2022) [28]. In order to detect the transposon sequences in the Canu contigs of the Horezu_LaPeri genome and to map the insertions relative to the reference genome, we harnessed Workflow 1 (WF1) and Workflow 2 (WF2) of the GA_v2 software, which are detailed elsewhere [21].

### 2.4. Multiple Alignment of KP Elements

A distinct adapted workflow was executed in GA_v2 by aligning the complete sequence of the P-element against the Canu contigs. Based on the coordinates defining the ends of alignments involving P elements, the sequences of P and KP elements were extracted using SeqKit v2.31 (https://github.com/shenwei356/seqkit, accessed on 16 December 2022) [29]. Then, the KP elements were multiple aligned using ClustalX v2.1 (http://www.clustal.org/, accessed on 1 February 2023) [30] with default parameters. To generate a consensus sequence for the KP element based on the ClustalX output, we used em_cons from EMBOSS package version 6.6.0.0 (https://emboss.sourceforge.net/, accessed on 1 February 2023) [31]. The consensus sequence of KP element has been deposited at GenBank and it is available under the accession number OQ693612.

### 2.5. GO Enrichment Analysis

Functional prediction analysis was performed using FlyEnrichr (https://maayanlab.cloud/FlyEnrichr/, accessed on 17 February 2023) [23] under default parameters. We analyzed two sets of genes, namely the genes potentially affected by the insertions specific to the Horezu_LaPeri genome (non-reference insertions), and also all of the genes hit by DNA NTs insertions in this genome.

### 2.6. Global Evaluation of Transposable Elements

For the global evaluation of the transposons landscape in the Horezu_LaPeri genome we used the LoRTE v1.2 (http://www.egce.cnrs-gif.fr/?p=6422, accessed on 24 February 2022) [20] and RepeatMasker v4.1.2 (https://www.repeatmasker.org/, accessed on 26 February 2022) [22] applications. Initially, the nanopore reads used to generate the Canu assembly were loaded into LoRTE along with the reference genome (release 5) and with associated transposon annotations (the only ones available for r5 and required for running the program). The plot showing the DNA NTs identified using LoRTE was generated using RStudio v1.3.959 (https://posit.co/, accessed on 26 February 2022) [32].

To run RepeatMasker, we used rmblastn version 2.11.0+ and the NTs library generated for Canu assembly with RepeatModeler v2.0.2 [33]. The latter integrates several repetitive sequence recognition algorithms—RepeatScout [34], RECON [35] and Tandem Repeats Finder (TRF) [36]— and is designed to build a library of repetitive elements for an assembly of interest. Consecutive to the identification of repetitive sequences, they were classified using the RepeatClassifier application, integrated in the RepeatModeler package. Landscapes of relative NT activity using Kimura distances [37] were generated running the calcDivergenceFromAlign.pl and createRepeatLandscape.pl scripts of the RepeatMasker package on .align files obtained by masking repetitive elements with RepeatMasker.

### 2.7. PCR Validation

For the P-element insertions bordered by identical TSDs, we performed a PCR validation of their genomic localization. The primers were designed with the Primer-Blast [38] application. The primers sequences, their genomic targets flanking the P-element insertions and the theoretical size of the expected amplicons are available in Supplementary Table S1. For TIRs regions of the P-element, we used MM11 primer (Supplementary Table S1) designed by Matyas Mink, University of Szeged, Hungary.

The PCR reactions were performed in a total volume of 25 μL containing 12.5 μL of Q5 High-Fidelity 2X Master Mix (NEB), 9 μL of Nuclease-Free Water, 1.25 μL of each primer pair (10 μM) and 1 μL of 39.5 ng/μL template DNA. Amplification was conducted under the same parameters except for two putative P-element insertions in *nolo* and *CG7991* genes, for which we used a double volume of MM11 primer. The amplification program was: initial hot start (30 s, 98 °C) and 30 cycles with denaturation (7 s, 98 °C), annealing (20 s, 61 °C), elongation (10 s, 72 °C), with a final extension of 2 min at 72 °C. The amplicons were run in a 1% agarose gel at 55 V.

## 3. Results

In order to detect and map the DNA transposons in the Horezu_LaPeri genome, we loaded in GA_v2 the canonical sequences of the class II NTs downloaded from the GitHub repository bermanlab/drosophila transposons [28]. Table 1 contains details about the DNA transposons analyzed in this work, such as their size, TIRs length and the number of annotated copies in the reference genome (r6.48) according to FlyBase. NTs such as looper1, FB, hopper2, INE-1 and Tc1-2 were not included in our analysis because FlyBase lacks data regarding their length and TIRs. The 1360 DNA NT was also not included due to its high frequency in the reference genome (~500 copies) and because of some erroneous annotations in FlyBase (i.e 1360{}6315, an annotated element of 1 bp length, found at genomic coordinates 2L:23,450,886..23,450,886).

The TIRs of each transposon sequence were extracted along with an extended 100 bp internal sequence (in order to differentiate between the 5′ and 3′ ends of the NT) and used as queries in WF1 of the GA_v2 tool. In this first stage of the mapping procedure, each transposon TIR defined as a query was aligned and had several significant hits in the Canu contigs collection loaded on the Genome Database. The significant alignments were exported in tabular form, providing the coordinates of the contigs where the TIRs sequences were identified. GA_v2 runs with bash scripts that allow for the extraction of TIRs from contigs along with their flanking sequence (the sequence in the immediate vicinity of the transposon), which is elemental for the mapping of each NT. We collected 3000 nucleotides long flanking sequences using parameters described elsewhere [21]. The extracted sequences are represented as Junction Queries (JQs) because they represent genome–transposon junctions and were used as queries in WF2 from GA_v2. WF2 involves the effective mapping of NT insertions using JQs, the reference genome of *D*. *melanogaster* as a Genome Database and the canonical sequence of an NT of interest as a Transposon Database. We used the reference genome version r6.48 downloaded from FlyBase along with the annotations of genes and transposons for a better evaluation of the genomic context. To map DNA transposons, JQs for reference genome alignments were generated for each NT. In the graphic interface of GA_v2, the most significant alignment was either completely blue (in the case of a conserved insertion), or blue-red (indicating a specific insertion in the Horezu_LaPeri genome). The conserved insertions are present both in the reference and in the analyzed genome and are either annotated or not by FlyBase. We also found cases of mapping ambiguities and unresolvable insertions. 

Table 2 lists the total number of mapped NTs insertions, mentioning the number of insertions present only in the Horezu_LaPeri genome, the number of conserved insertions as well as the number of ambiguous and unresolvable cases for each NT. The total number of mapped insertions was calculated by adding the Horezu_LaPeri-specific insertions to the conserved insertions.

The data generated with GA_v2 were manually curated and inventoried in two large tables (Table 3 and Table S2) that contain details about the type of NT, the host chromosome and the coordinate where it is located, the hit/close genes and the type of insertion (conserved or specific to the Horezu_LaPeri genome). It is known that in *D. melanogaster*, regulatory elements can be located tens of kilobases away or in the proximity of the gene they regulate [39,40,41]. Therefore, we included in our analysis the genes tagged by relatively close transposon insertions (genes whose sequence was found in the extracted flanking sequence) since their activity/expression could be affected. In Table 3 are mentioned only the DNA transposon insertions specific to the Horezu_LaPeri genome, while Supplementary Table S2 contains conserved insertions which are annotated or not by FlyBase. All of the NT insertions located in the Y chromosome and in polytenic regions 20, 40, 41, 80 and 81 of the *D. melanogaster* are marked with an asterisk (*). Each of the DNA transposons mentioned in Table 1 was mapped using GA_v2 to obtain a transposon insertion coordinate relative to the reference genome. In some cases, the mapping was ambiguous, either because of the repetitive nature of the genomic region where the NT was inserted or because of the limited size of the contigs. Thus, some of the hobo, hopper, pogo, mariner2 and S-element insertions were resolved by extending the flanking sequence (where possible) to 6000 nucleotides. Regarding the P-element, we applied both the workflows from the GA_v2 package, as well as an adapted workflow consisting in the alignment of the integral sequence of the P-element in WF1 and in the extraction of the integral P-element sequences from the contigs, in order for it to be submitted to multiple alignment.

The annotated conserved insertions are specified by the NT element FlyBase identifiers in Supplementary Table S2. The unannotated conserved insertions are referred by the genomic coordinate where the element is located in the same manner the NT insertions specific for the Horezu_LaPeri genome that are mentioned in Table 3. An example of an unannotated insertion which is actually present in r6.48 is the hobo insertion from genomic coordinate 2R:13,677,674 in the *tei* gene. Whenever the mapping was not possible or it was limited just to chromosomal arm resolution, either an “Unresolvable” or “Most probably in” flag was used. 

We inventoried two clusters of Bari1 elements in the Horezu_LaPeri genome. A major cluster known to be located in the h39 region of chromosome 2R [42,43,44,45,46] was inferred using eight contigs from the Canu assembly. The second one is similar to a minor cluster previously mapped on the X chromosome [44,45]. These Bari1 clusters are still unannotated in FlyBase, although they are reported in several papers [42,43,44,45,46]. According to our mapping data, the Bari1 complex identified in the 2R chromosome of Horezu_LaPeri spans the genomic interval 2R:180,603..287,454, pertaining to polytenic region 41A. Additionally, a detailed analysis of contig 2099 revealed that the small Bari1 cluster identified in the X chromosome contains three full-sized copies and one truncated Bari1 element, located in a rDNA repeats region of cytogenetic band 20F4, upstream of the *28SrRNA:Ψ:CR45855* pseudogene.

**Figure 1:**
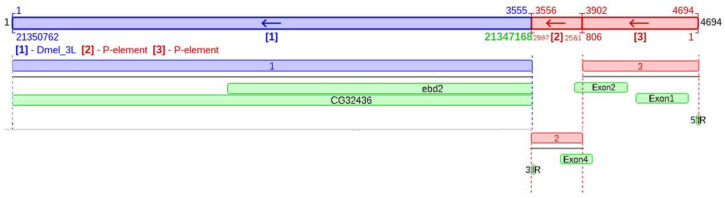
Example of visualization of genes and transposons annotations in the GA_v2 graphic interface. The reference genome is marked with blue, and the sequence of the transposable element is marked with red. The green boxes show the annotations, revealing that this insertion of the P-element has an internal deletion between positions 806 and 2561 (KP element) in the overlapping genes *ebd2* and *CG32436*.

GA_v2 allows for the annotation of a whole genome sequence, but also permits the annotation of shorter sequences such as genes or transposons. Since one of our focuses was the analysis of the P-element, we added “features” to the canonical sequence as TIR regions and transposase exons to evaluate sequence polymorphisms between P-element copies from the Horezu_LaPeri genome and the P-element’s canonical sequence. An example of visualizing the genomic context and specific annotations of the P-element is shown in Figure 1, which depicts a KP element (deletion between 806 and 2561 positions) inserted in the overlapped genes *ebd2* and *CG32436*.

**Figure 2:**
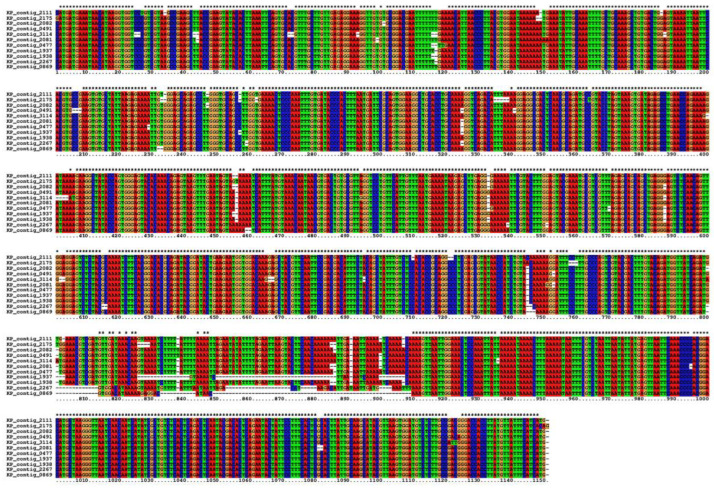
Multiple alignment of 11 KP elements extracted from Canu contigs of the Horezu_LaPeri genome using ClustalX v2.1 application. The stars indicate the number of completely conserved columns in the multiple alignment.

Consecutive to this analysis, a total of 16 P elements were identified in the Horezu_LaPeri genome. Out of these, 11 had the specific internal deletion of the KP element (806–2561, with an offset of a few nucleotides). In order to obtain a consensus sequence, KP element sequences were extracted from the contigs using SeqKit and multi-aligned with the ClustalX application (Figure 2). Two other insertions had internal deletions but at different positions comparative to the KP element (899–2368 and 656–1146, respectively). Three more P-element insertions were detected based on a single TIR, but with an incomplete sequence (<800 bp) due to sequencing/assembly limitations.

The consensus sequence of the KP element was generated using em_cons and has an internal deletion between 805 and 2561 coordinates, similar to the ones described in other studies [6,7,8,9,10]. This KP consensus sequence was deposited in NCBI with the GenBank accession number OQ693612.

Some of the P-element insertions in the Horezu_LaPeri genome were also verified with the PCR technique. Only the insertions with TSDs (Supplementary Table S3) were PCR checked, and the obtained amplicons were migrated in agarose gel (Supplementary Figure S1). Some of the tested insertions were identified using the Canu assembly analyzed in this paper, while three other P-element insertions (in *Ac13E*, in *retn* and near *stv*) with identical TSDs were identified in alternative assemblies described elsewhere [21,24]. The PCR primers are specific to the sequences that flank the insertions of the P elements (Supplementary Table S1) and together with the MM11 primer (specific for TIRs of the P-element) were used to amplify the genome–transposon junction, thus testing the presence of the insertions. 

Out of the 15 P-element insertions tested via PCR, we confirmed eight, namely insertions detected in *ebd2*, *CG32436*, *Ac13E*, *retn*, *nolo*, *CG7991* and close to *CG5555* and *stv* genes. Attempts to amplify the rest of the P-element insertions with PCR parameters and protocol adjustments were also unsuccessful, most likely due to a suboptimal primer design, sequencing/assembly issues or inaccurate mapping.

Besides P elements, we identified multiple non-reference DNA NTs insertions (particular to the Horezu_LaPeri genome). The most frequent ones are hobo (26), transib1 (19), pogo (18) and 13 hopper insertions. An interesting aspect is that most of these insertions are located in genes or in lncRNAs, and only a few of them are located in non-coding regions. On the other hand, some of these transposons are located in insertional hotspots, arbitrarily defined herein as sites containing at least three NT insertions according to annotation data available in FlyBase. This may suggest that even after recent transposition events, some transposons have an affinity for certain regions known to host NTs insertions. For Bari2, S2, transib3 and transib4 elements, we did not identify any non-reference insertions. In addition to Horezu_LaPeri-specific insertions, we mapped many conserved insertions. Most of the NTs reported with common sites to the reference were S-element (74), HB (46), Tc1 (43), mariner2 (35) and transib2 (34). 

**Figure 3:**
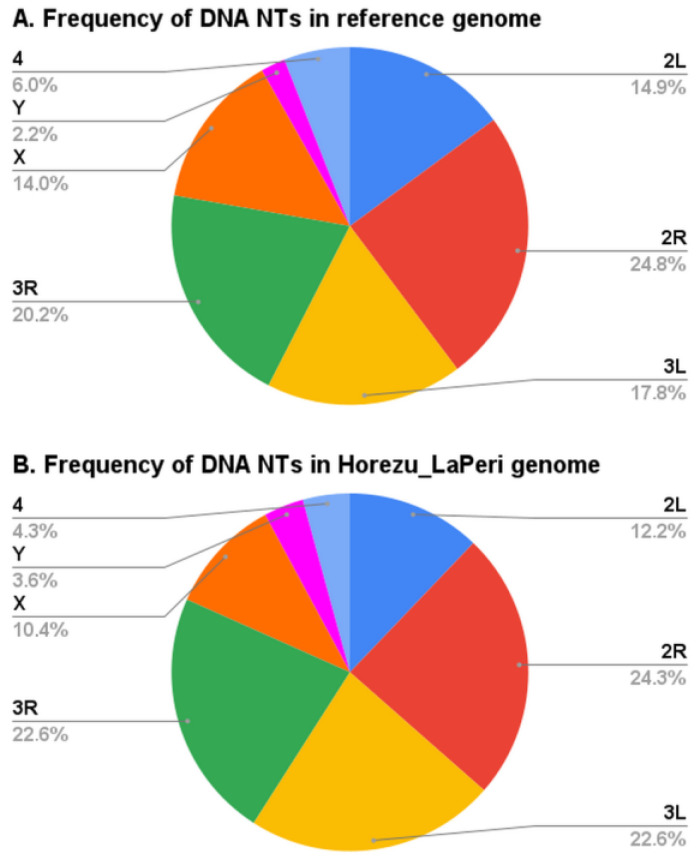
Frequency distributions of considered DNA NTs in reference genome (**A**) of *D. melanogaster* (r6.48) and in the Horezu_LaPeri genome (**B**). All DNA NTs from the Horezu_LaPeri genome were identified using GA_v2 software. DNA NTs from the reference genome include both the FlyBase-annotated NTs and the unannotated NTs identified with GA_v2 in this study.

Overall, we mapped with GA_v2 a total number of 469 DNA NTs, 120 being specific to the Horezu_LaPeri genome (non-reference) and 349 being also present in the reference genome. We found that out of the 349 conserved insertions, 163 are still not annotated by FlyBase. Furthermore, we counted the frequency per chromosome of the analyzed DNA NT insertions in the Horezu_LaPeri genome and in the reference genome (Figure 3). Regarding the Horezu_LaPeri genome (Figure 3B), most of the NT insertions are in the 2R chromosome (*n* = 114, 24.3%), followed by 106 insertions in 3L (22.6%), 106 insertions in 3R (22.6%), 57 insertions in 2L (12.2%), 49 insertions in X (10.4%), 20 NTs in chromosome 4 (4.3%) and only 17 in Y (3.6%). Frequency analysis of the same DNA NTs, annotated and unannotated in the reference genome, showed that the distribution of NTs in the Horezu_LaPeri genome is similar to their distribution in the reference. For example, in the reference genome, the percentage of DNA NTs in chromosome 2R is 24.8%, 14.9% in chromosome 2L and 20.2% in chromosome 3R.

Data analysis of GA_v2 results was completed with the association of GO terms and the prediction of functional interactions for hit/close genes. In order to evaluate the potential impact of DNA NT insertions on gene function, we used FlyEnrichr for functional prediction analyses. We initially investigated all the genes associated with insertions particular to the Horezu_LaPeri genome (presented in Table 3). Then, we performed the functional annotation of two gene sets associated with DNA NTs (the genes specified in Table 3 and, respectively, the genes near/affected by conserved insertions depicted in Supplementary Table S2). GO enrichment analysis of hit/nearby genes affected by NTs in the Horezu_LaPeri genome indicates that some of these genes could be involved in biological processes associated with adaptation to the environment and with responses to different stimuli. Significant and relevant GO terms are mentioned in Table 4, along with the corresponding genes and P values. In Table 4, the first 12 mentioned GO terms in the A section were identified using only Horezu_LaPeri non-reference insertions (Table 4A), while the B section of the table includes GO terms identified using both Horezu_LaPeri reference and non-reference insertions.

The main strategy of GA_v2 is based on aligning each query (represented by a transposon-flanking sequence) to the databases (the reference genome and the canonical sequence of a given NT). Other tools dedicated to the analysis of NTs rely on different strategies and are pre-loaded with a whole NTs database for the purpose of a global analysis. For a global evaluation of the DNA NTs in the Horezu_LaPeri genome, we used the LoRTE v1.2 and RepeatMasker v4.1.2 applications, which take into account both class I and class II transposons from the *D*. *melanogaster* genome. 

**Figure 4:**
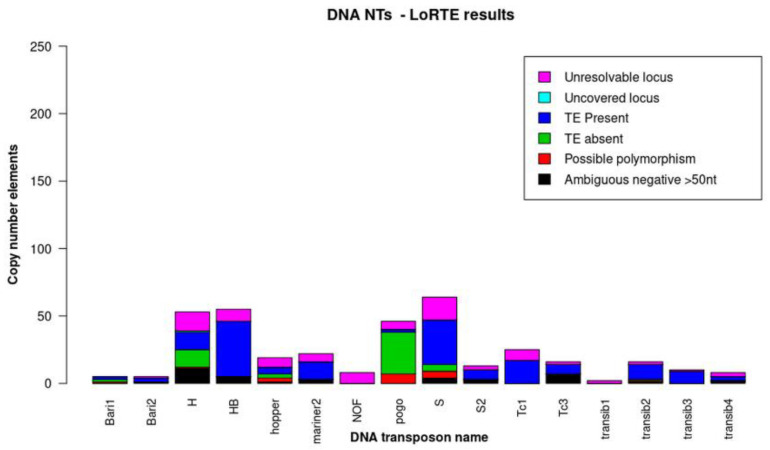
The frequency of DNA NTs obtained with LoRTE for the ONT reads generated via sequencing of the Horezu_LaPeri genome. The *y*-axis shows the number of copies of NTs, while on the *x*-axis are represented the transposons’ families. The color code is associated with the annotations generated using LoRTE for each analyzed element.

LoRTE compares the NT insertions identified in long reads with the annotated NTs from the reference genome (release 5) and computes the number of new and conserved insertions. Thus, we analyzed the Horezu_LaPeri genome using all the NTs from the LoRTE database, but we represented graphically only the DNA transposons indicated in Table 1. The P-element was implicitly excluded because it is not found in the reference genome. The results obtained with LoRTE are shown in Figure 4. 

Overall, LoRTE covered 99.95% of the NTs inventoried in the database. Out of them, seven new insertions specific to the Horezu_LaPeri genome were identified (Supplementary Table S4). Two of these are instances of the M4DM element from the Transib family at coordinate 2L:20,969,629, in an intron of *CG42238*, with coverage = 13 and at X:18,356,645, in a repetitive element INE-1{}4916 with coverage = 4. A third insertion is a Transib2 element located in 3R:14,049,171 in a noncoding region with a coverage of 7. This insertion was also identified with GA_v2, following WF1 and WF2 on Transib2 elements. The remaining four are insertions of LTR- or LINE-type elements (one is an LTR element in an intron of *CG1358* and three are LINEs— one in the exonic region of *yuri*, one in an intron of *SKIP*, and one in a noncoding region).

**Figure 5:**
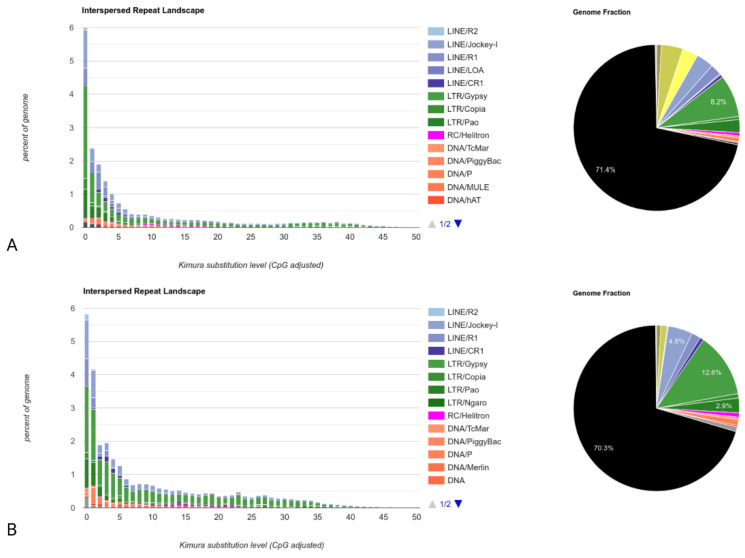
NTs’ landscape divergence diagrams computed with RepeatMasker for: (**A**) the proportion of repetitive elements in the reference genome of *D*. *melanogaster* (dm3); (**B**) the total number of repetitive elements in the Horezu_LaPeri genome. The *y*-axis represents genome coverage for each transposon type, and the *x*-axis represents Kimura distances.

In contrast to LoRTE, RepeatMasker scans an assembly to identify repetitive elements. This global analysis consists of scanning the contigs of the Horezu_LaPeri genome for both DNA transposons and retrotransposons (class I transposable elements). First, we built a de novo repeat library with RepeatModeler v2.0.2 (using RECON, RepeatScout, TRF and RMBlast) of the Canu assembly of the Horezu_LaPeri genome. Subsequently, this assembly was masked by RepeatMasker v4.1.2 (RM-BLAST mode) using the NT custom library. To assess the NTs’ degree of activity, the Kimura parameter of divergence for the NTs was calculated between the consensus sequence of each NT and all identified copies in the assembly. The NTs’ divergences were calculated using the RepeatMasker built-in tool calcDivergenceFromAlign.pl, and the NTs’ landscape divergence plot (Figure 5) was generated using createRepeatLandscape.pl script. Figure 5 shows the genome coverage (*y*-axis) for each type of transposon (LINEs, LTRs, DNA NTs) in the reference genome of *D*. *melanogaster* dm3 (Figure 5A) versus the Canu assembly of the Horezu_LaPeri genome (Figure 5B). Transposons are grouped according to Kimura distances (*x*-axis) relative to their consensus sequences. Clustered NT copies on the left of the graph do not deviate much from the consensus element sequence and most likely correspond to recent copies, while sequences on the right of the graph represent degenerate copies that differ substantially from the consensus sequences. Each pie chart shows the total unmasked DNA (black color) and the content of all NTs in the reference genome (Figure 5A) and in the Horezu_LaPeri genome (Figure 5B).

The results generated with RepeatMasker by evaluating the NT content of the Horezu_LaPeri genome suggest that the proportion of NT is 29.7% for the Canu assembly, very close to 28.6%, a value calculated with RepeatMasker for the reference genome [47]. 

## 4. Discussion

The main aim of this study was to assess the DNA transposons landscape of the *D*. *melanogaster* Horezu_LaPeri genome. A general conclusion is that the whole genome sequencing by ONT can provide enough information to detect NTs in the genome of a natural population of *D*. *melanogaster*.

All of the contigs generated with the Canu application were initially used in a pair-wise alignment analysis with the GA_v2 application, and a total number of 469 DNA NTs were mapped in this genome. Out of them, 120 were specific (non-reference) insertions, 349 were conserved insertions (from which 163 were unannotated by FlyBase), 7 were ambiguous mapped insertions and 26 cases were unresolvable. These insertions were identified by aligning the DNA transposon sequences to Canu contigs, then the JQs were extracted and used to map the insertions versus the reference genome of *D*. *melanogaster*. It is noticeable that this genome contains no less than 120 non-reference insertions. As inferred from bioinformatic predictions analyses, some of them are prone to be involved in the adaptation to the environment variables.

Furthermore, the ONT sequencing allows for the obtaining of long reads and (consecutive to the assembly step) of contigs which may contain sequences specific for the heterochromatin regions. According to the release 5.1 annotations of *D. melanogaster* heterochromatin [48], there are estimated to be at least 230 heterochromatic genes, but we found only 214 heterochromatic genes at https://flybase.org/reports/FBrf0188763 (accessed on 4 May 2023). Further data refinements kept only 148 current genes, while the rest of the 66 genes are nowadays reported as withdrawn (according to www.flybase.org, accessed on 4 May 2023). All of the considered heterochromatic genes are located in polytenic regions 20, 40, 41, 80 and 81, or in the heterochromatic chromosome Y. Out of the 229 genes affected by DNA NTs insertions in the Horezu_LaPeri fruit fly strain, we identified insertions in 80 heterochromatic current genes, which are hit by 170 (154 conserved + 16 non-reference) insertions of DNA NTs. Conversely, the rest of the 149 non-heterochromatic genes are hit by a total of 137 DNA NTs insertions. Therefore, the heterochromatic genes are hit by an average of 2.12 insertions/gene, while the non-heterochromatic genes are hit, on average, by 0.91 insertions/gene. These data reveal that the incidence of DNA NTs insertions in heterochromatic genes is 2.32X relative to the non-heterochromatic genes in the Horezu_LaPeri genome, a value confirming former observations that the introns of the heterochromatic genes are enriched for transposon insertions [49,50]. Additionally, we found a total of 162 DNA NTs insertions located in various non-coding regions. Remarkably, 110 (17 non-reference + 93 conserved insertions) of them (67.9%) are located either in the Y chromosome, or in the 20, 40, 41, 80 and 81 cytogenetic regions (NT hotspots, natural transposons or intergenic regions marked with * in Table 3 and Table S2). From a total of 469 DNA NT insertions detected using our mapping workflow applied on the Canu assembly of the Horezu_LaPeri genome, 349 are conserved between the two genomes considered herein. Notably, more than two-thirds (247/349) of the conserved insertions between Horezu_LaPeri and the reference r6.48 genomes are located in heterochromatic regions. Out of these conserved hits, 154 insertions are located in or close to various genes, while the rest of the 93 insertions hit other NTs or non-coding regions. Regarding the 120 non-reference insertions, a total of 33 are located either in the Y chromosome, or in polytenic regions 20, 40, 41, 80 and 81 (16 hits inside or close to different genes, and 17 insertions are associated with NTs or non-coding regions). These data reveal a high degree of conservation for many insertional events of DNA NTs during the evolutive process.

Considering that NT insertions often cause gene disruption as a consequence of the transposition events, our results support the hypothesis that transposon insertions in euchromatin are less tolerated compared to those in heterochromatin [51]. Most probably, the accumulation of NTs in heterochromatin regions could be explained by the fact that deleterious effects of NTs are somehow milder or even absent in these regions. Overall, the DNA NTs landscape resulted from the Canu assembly of the Horezu_LaPeri genome suggests that pericentromeric heterochromatin is a transposon-rich region with multiple NTs mapped and residing therein. Our results are in accordance with the reported data [52,53,54], showing that the pericentromeric heterochromatin is one of the preferential sites for various transposons, which contribute to the expansion of heterochromatin blocks through evolutionary time, starting from local accumulations of NTs.

Since the P-element is one of the most intriguing transposons present in the natural populations of the *D*. *melanogaster* genome, we approached a detailed analysis of this NT in the Horezu_LaPeri genome. Our results reveal that this local population was exposed to the invasion of the P-element, which is a relatively recent event (early 20th century) [55,56]. The presence of the P-element in the fruit flies collected from Horezu, Valcea and Romania; as well as in other populations collected from different regions in Asia, Africa, Australia, Europe, South and North America [57,58,59], reinforces the conclusion that the P-element has a ubiquitous presence nowadays. This is an interesting aspect, as the fruit fly strain used in this study was collected from a relatively isolated geographic region. Distinctive from other natural populations present in various regions of Europe (France, Russia), the Americas, Australia and Africa, we found no full-sized P elements in the population analyzed in this study. Populations that lack any full-sized P elements have also been reported in Cygnett, Australia [60] and in Chichi Jima, Japan [61], but in very low proportions comparative to the worldwide populations where KP elements and complete P elements predominate. In a recent study that proposed a model for the invasion route of the P-element in natural populations of *D*. *melanogaster*, it is mentioned that the number of autonomous P elements decreased in Europe from west to east. As an example, very few full-sized elements were detected in some Ukrainian populations [61]. So far, there were no reported data about *Drosophila* natural populations in Romania, and the only reported information is described in the present article and, briefly, in a related paper [21]. Our data appear to support the hypothesis that P-element invasion may have “died down” in the East of Europe consecutive to a loss of autonomous P-element insertions. The putative model of P-element invasion suggests that the transposon spread from Africa to Europe, then starting from France, it invaded the populations of Spain and Eastern Europe [61].

Our bioinformatics analysis indicates that all P elements of the Horezu_LaPeri genome are non-autonomous as we did not find any P elements liable to produce transposase. We therefore presume that the population of *D*. *melanogaster* Horezu_LaPeri is in a state of equilibrium regarding the P-element transposition dynamics. Our analyses match the results obtained by Black et al. in 1987 [10] regarding *D*. *melanogaster* populations in Europe, pointing to the fact that these populations contain few integral P elements and that their frequency decreases gradually from France to central Asia, where many copies of KP elements predominate. 

The only consensus sequence reported for the KP element was obtained based on two elements with identical sequences that had a length of 1154 bp, with an internal deletion defined by coordinates 808 and 2560 of the P-element reference sequence [10]. Conversely, the consensus generated in our study is based on the multiple alignment of 11 different KP insertions and consists of a sequence of 1139 bp, which contains a deletion defined by the coordinates 805 and 2561 of the canonical sequence of the P-element. Using the PCR technique, we validated the presence of some P/KP elements in the Horezu_LaPeri genome inserted within or in the vicinity of genes. We verified only the P-element insertions with intact TSDs (Supplementary Table S3). We also mention in Table S3 the exonic/intronic region (when applicable) hit by the P-element insertions.

In addition to the P-element analysis, we located at the nucleotide level 16 other DNA NTs in the Horezu_LaPeri genome, namely Bari1, Bari2, HB, hobo, hopper, mariner2, NOF, pogo, S-element, S2, Tc1, Tc3, transib1, transib2, transib3 and transib4. This mapping data enabled us to compile a list of genes prone to be affected by some of these insertions, which proved to be a valuable support for our further functional prediction analyses described in the present article. We noticed that the global distribution of the DNA NTs in the Horezu_LaPeri genome is highly similar to the one in the reference genome (Figure 3). As expected, most of the DNA transposons from the Horezu_LaPeri genome are located in the large chromosomes, namely 2 (36.5% insertions) and 3 (45.2% insertions). While the percentage of DNA NTs in the X chromosome is 10.4%, fewer insertions were identified in chromosomes Y (3.6%) and 4 (4.3%). This aspect reflects once again the robustness of the NT annotation workflows run by GA_v2. 

**Figure 6:**
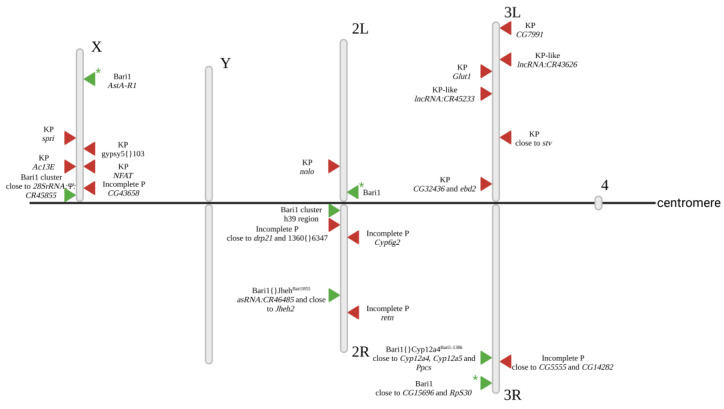
The distribution of the P-element and Bari1 NTs in the Horezu_LaPeri genome. The red triangles represent the insertions of the P-element, while the green triangles represent insertions of the Bari1 element, an NT found in both genomes. The green triangles with an asterisk (*) stand for Bari1 insertions which were detected only in the Horezu_LaPeri genome. P-element insertions in/near to *Ac13E*, *retn* and *stv* genes were mapped using an alternative assembly. Created with BioRender.com (accessed on 15 May 2023).

The distribution of DNA NTs analyzed in this paper is represented as tables, generated following their actual mapping in the Horezu_LaPeri genome. Since their number is relatively large (of the *order of hundreds*), we chose to graphically represent two examples of NTs, one reference NT (Bari1) and one non-reference (P-element) NT. The distribution of Bari1 copies in the Horezu_LaPeri genome versus the reference r6.48 is depicted in Figure 6. Each copy of Bari1 is represented by a green triangle; asterisk (*) tags highlight copies specific to the Horezu_LaPeri genome. In parallel, we represented the distribution of the P-element (red triangles), a transposon which is by default absent in the reference genome.

In the attempt to verify the mapping data for the P-element, we obtained the expected PCR amplicons for only 8 out of 16 cases (Supplementary Figure S1). The PCR data confirm that a P/KP element is located in or close to *ebd2*, *CG32436*, *Ac13E*, *Glut1*, *retn*, *nolo*, *CG7991*, *CG5555* and *stv* genes (*ebd2* and *CG32436* are overlapped genes). According to FlyBase, the *nolo* gene is a rich hotspot for various NTs. 

A comparative inquiry against 90 other natural populations of *D. melanogaster* [62] revealed that all of the three Bari1 insertions depicted in Figure 6 (marked with green and *) are specific only for the Horezu_LaPeri genome. Particularly, the Bari1 element located in the 2L chromosome is inserted into a pericentromeric heterocromatin region (40F7 cytological band, genomic coordinate 2L:23,430,916). As discussed elsewhere, DNA NTs are abundant in the constitutive heterochromatin, raising questions about the roles of these insertions in the structure and functions of the centromere [51]. Notably, the origin of two conserved Bari1 clusters located in the heterochromatic regions of chromosomes X and 2 of *D. melanogaster* is interrogated [51]. A deeper analysis of specific contigs from the Canu assembly, performed with an alternative GA_v2 mapping approach, revealed that both Bari1 clusters are also present in the Horezu_LaPeri genome. This strong positive selection of these Bari1 tandem repeats most probably reflects their key roles in the functional architecture of the centromere in *D. melanogaster*. Conversely, we found no clusters for two recent DNA NTs invaders, namely the hobo and P-element, yet they were reported before for various natural populations of *D. melanogaster* [63]. The absence of hobo and P-element clusters in our Canu assembly may be explained by a rather recent occurrence of these two DNA NTs in the Horezu_LaPeri genome, so clustering insertional events had not yet occurred. This hypothesis is supported by the fact that this strain was derived from fruit flies collected from a rather isolated geographical location of Romania (see Section 2). 

For *kay*, *Glut1* and *Pka-C1* genes, which are potentially affected by insertions of the P-element according to our bioinformatics data, FlyBase reports insertional alleles conducting to an abnormal temperature response phenotype. According to FlyBase, the alleles for *Glut1* and *kay* were obtained with artificial derivatives from the P-element. Additionally, a P insertion identified in an alternative assembly of the Horezu_LaPeri genome [21] hits the *Cyp6g2* gene. For this gene, in FlyBase it is reported as a single biological process based on experimental evidence (inferred from mutant phenotype), namely the response to insecticide [64]. 

Global enrichment analysis of the genes close to or hit by insertions of NTs in the Horezu_LaPeri genome (Table 3 and Table S2) revealed that many of the insertional alleles may have a potential adaptive role. Phenotype and GO enrichment inquiries identified significant terms such as temperature compensation of the circadian clock, temperature response defective, response to alcohol, sensory perception of touch, increased fecundity, radiation resistance and circadian rhythm defective (Table 4). Many NT insertions specific for the Horezu_LaPeri genome are located in or nearby genes involved in different cellular processes crucial for adaptation to the environment. For example, the overrepresented GO term of temperature compensation of the circadian clock defines a process by which organisms can keep their circadian rhythm constant, even if the temperature of the environment varies (GO:0010378). Therefore, it is expected that mutant alleles of the genes involved in this process (*bru3*, *Pde6*, *Pka-C1*, *CG8910*, *CG32085*, *Pde1c*, *GABA-B-R1*, *Dgk*, *CG3453)* can lead to changes in the circadian rhythm and in the adaptation response to environmental temperature changes. If these alterations prove to be advantageous under certain environmental conditions, they might be further selected and propagated in a natural population of *D*. *melanogaster*. One of the overrepresented phenotypes is “temperature response defective”, indicating a potential adaptation to the environment of this natural population of *D*. *melanogaster* via the natural selection of mutations more likely to survive consistent temperature fluctuations. Therefore, insertional mutations in genes leading to a “temperature response defective” phenotype (*CG8910*, *GABA-R1*, *mub*, *CG34353*) may be positively selected and then spread in the fruit fly local populations.

We also found that some NT insertions may impact the expression of genes that regulate neurotransmission (*Pka-C1*, *cpx*, *Frq2*, *Nlg4*, *Dys*) and can affect the communication between neurons and the regulation of some physiological processes, such as sleep, food intake, social behavior and the response to external stimuli [65,66]. Transposon insertions in genes involved in the regulation of muscle tissue development (*Mef2*, *Kank*, *Ten-m*, *CadN*, *beat-Ia*, *Egfr*, *Dys*) may as well contribute to environmental adaptation in *D*. *melanogaster*. Changes in the expression of regulatory genes can affect muscle development and performance, which may have consequences for the ability to adapt to environmental conditions [67,68]. Additionally, transposon insertions in genes involved in R7 cell differentiation and rhodopsin biosynthesis (*Ten-m*, *CadN*, *rau*, *Egfr*) can influence the ability of a natural population of *D*. *melanogaster* to adapt to the environment by changing the sensitivity of the eyes to light or by influencing other behavioral or physiological characteristics [69,70]. It is possible that transposon insertions in genes involved in cellular metal ion homeostasis (*RhoGEF3*, *Pde6*, *Pde1c*, *CG8910*, *Glut1*, *GABA-B-R1*, *Dgk*, *cpx*, *CG34353*) contribute to environmental adaptation in *D*. *melanogaster*, as these genes can affect important physiological processes such as metal ion transport across cell membranes as well as their storage and disposal. In addition, metal ions can play an important role in various biochemical processes, including protein synthesis and energy metabolism, which are critical for environmental adaptation [71].

Although class I NTs are not the main subject of our study, we consider significant to mention that LoRTE analysis identified some new NT insertions of Gypsy, Jockey and Transib families, which are specified in Supplementary Table S4. Two of the novel insertions identified in the Horezu_LaPeri genome are located in the introns of the *CG1358* and *SKIP* genes that have been reported to be involved in circadian rhythm [72,73,74]. The *SKIP* gene has a role in the sensory perception of smell, and insertions of natural NTs in the *SKIP* gene were reported to have an adaptive role [75]. A Transib element was identified inside a repeat region (INE-1{}4916) located upstream of the *Kairos* gene, which has also been reported to be involved in circadian rhythm. In 2021, the analysis of *Kairos* revertants showed a regulation of the circadian rhythm, suggesting that this gene has an important role in regulating locomotor activity behavior under constant dark conditions [76]. Another Transib element was identified in the intron of the *CG42238* gene with an unknown function. Overall, all these insertions suggest a potential regulation of the circadian rhythm and an adaptation to the biological, chemical and physical factors of the environment specific to the Horezu region.

Starting from Kimura distance-based copy divergence analysis of NTs of the Horezu_LaPeri natural population (calculated with the RepeatMasker package), we estimated the dynamics and the chronological distribution of transposons insertions. In Figure 5, NT copies located to the left of the plot have low Kimura values and potentially correspond to recent events, while older NT copies are placed on the right of the plot. The transposable elements landscape obtained for the Canu assembly is highly similar to the reported RepeatMasker analysis of transposable elements from the dm3 reference genome [47]. The graph follows an L-shaped pattern, suggesting the recent activity of NTs [77,78], especially of class I elements (Kimura values close to 0). However, it should be noted that this is only an estimation, as the degree of divergence between identified NTs and their consensus sequences may be influenced by potential sequencing/assembly errors.

Although a more detailed analysis of various alternative annotations of the Horezu_LaPeri genome would bring minor data adjustments, we consider that the Canu assembly of the unfiltered reads offers a solid platform for portraying the landscape of DNA NTs of both euchromatic and heterochromatic regions. Our study is expected to contribute to a better understanding of the DNA NT dynamics and to the deciphering of the structure and evolution of the *D*. *melanogaster* genome. As no Romanian fruit fly strain was sequenced so far, we estimate that our study is useful for the characterization of natural populations of *D*. *melanogaster* in Eastern Europe.

## Figures and Tables

**Table 1:** DNA transposable elements analyzed in the Horezu_LaPeri genome (displayed in *alphabetical order*). The lengths of the NTs and their TIRs, as well as the number of annotated copies are reported according to FlyBase (release 2022_05).

DNA NT Name	NT Length (bp)	TIR Length (bp)	Copy Number in *D. melanogaster* r6.48
Bari1	1728	26	7
Bari2	1064	253	5
HB	1653	29–31	60
hobo	2959	12	60
hopper	1435	33	26
mariner2	912	29	23
NOF	4347	308	8
P-element	2907	31	0
pogo	2121	26	50
S-element	1736	234	187
S2	1735	233	16
Tc1	1666	26	31
Tc3	1743	43	19
transib1	2167	43	3
transib2	2844	42	27
transib3	2883	45	13
transib4	2656	40	8

**Table 2:** Total number of mapped/unmapped DNA transposable elements with GA_v2 in the Horezu_LaPeri genome relative to the *D*. *melanogaster* reference (r6.48). The total number of mapped insertions includes both the Horezu_LaPeri-specific insertions and the conserved ones. The unannotated conserved insertions (fourth column) are included in the conserved insertions (second column) and show the number of conserved insertions which are not annotated by FlyBase. The ambiguous insertions and unresolvable insertions columns were not taken into consideration for further analysis and refer to transposons that were detected but not mapped.

DNA NT	Horezu_LaPeri-Specific Insertions	Conserved Insertions	Total Mapped Insertions	Unannotated Conserved Insertions	*Ambiguous* Insertions	Unresolvable Insertions
Bari1	3	3	6	1	1	1
Bari2	0	4	4	2	0	0
HB	1	46	47	10	0	1
hobo	26	12	38	9	0	0
hopper	13	26	39	14	0	1
mariner2	1	34	35	16	0	0
NOF	1	2	3	1	0	0
P-element	16	0	16	0	0	0
pogo	18	4	22	0	1	2
S-element	12	74	86	39	2	10
S2	0	16	16	7	0	2
Tc1	1	43	44	15	0	3
Tc3	3	9	12	4	0	0
transib1	19	12	31	10	0	2
transib2	6	34	40	20	2	2
transib3	0	22	22	13	0	0
transib4	0	8	8	2	1	2

**Table 3:** DNA transposons insertions specific to the Horezu_LaPeri genome mapped with GA_v2 (displayed in *alphabetical order*). The chromosomal location, the genomic coordinates and the hit/close genes/NTs are reported for each mapped NT insertion. Insertions marked with an asterisk (*) stand for NTs located in various heterochromatic regions.

NT Name	Contig Number	Insertion Coordinate; Host Chromosome	Hit/Close Genes/NTs
Bari1	892	3,657,194; X	*AstA-R1*
	2963	20,848,451; 3R	close to *CG15696* and *RpS30*
	3495	23,430,916; 2L	-*
HB	2615	22,744,158; 3L	CG7369* NTs hotspot
hobo	37	11,731,193; 3R	*CG12594*
	142	8,265,913; 3L	*Dscam4*
	153	13,627,239; 3L	*bru3*
	166	31,134,735; 3R	*Gycβ100B*
	364	11,729,899; X	*inaF-A* *inaF-B* *inaF-C* *CG15221*
	429	16,058,145; 2L	*beat-Ia*
	1066	18,846,823; 3R	*Octα2R*
	1121	11,868,933; 2L	*Pde1c*
	2025, 2026	8,555,248; X	*IntS4*
	2056	12,963,046; 2L	-
	2063	13,826,443; 3L	-
	2110	13,678,976; 3L	*bru3*
	2111, 2421, 2422	14,043,231; 2R	*mam*
	2124	8,322,604; 3L	-
	2136	16,515,554; 3L	*CG43373*
	2166	17,551,994; 2L	-
	2167	19,461,109; 2L	-
	2316	17,384,934; 2R	*lncRNA:CR44344*
	2343	17,184,472; 2R	*lncRNA:CR44387*
	2484	20,235,449; 3R	*Nlg4*
	2673	5,866,909; 2L	*rau*
		5,649,715; 2L	*DIP-θ*
	2707	15,577,454; 2R	-
	2762	9,291,978; 3R	-
	2788	22,850,595; 3R	*lncRNA:CR43846*
	3145	16,137,749; 2L	*lncRNA:CR44871*
hopper	165	18,193,772; X	*Frq2*
	526	16,891,891; 2R	*CG8910*
	1264	22,559,246; X	1731{}3268* NTs hotspot
	1938	5,438,793; 3L	*DIP-δ*
	1989	20,939,569; 2L	-
	2020	27,859,138; 3R	*CG34353*
		27,974,479; 3R	*βTub97EF*
	2615	22,635,736; 3L	*CG14459*
	2675	21,846,566; 3L	*mub*
	2725	2,586,216; 2R	1360{}6340*
	2760	12,301,235; 3L	*lncRNA:CR44550*
	3122	24,568,086; 3L	-*
	3153	11,661,763; 3L	*CG32085*
mariner2	744	2,671,627; 3R	Pzl*
NOF	2967	19,470,432; 3R	*Dys*
P-element	477	29,765,960; 3R	close to *kay*
	491	21,347,168; 3L	*ebd2* *CG32436*
	869	10,547,695; X	*spri*
	1601	3,250,477; 3L	*lncRNA:CR43626*
	1937	959,438; 3L	*Glut1*
	1938	1,688,826; 3L	*CG7991*
	2081	7,489,940; 2R	close to *Coop*
	2082	21,697,891; 2L	*nolo* NTs hotspot
	2111	14,928,221; 2R	*Kank*
	2122	13,477,242; 3L	close to *CG10089*
	2175	289,069; 2R	-*
	2267	9,698,895; 2L	*Pka-C1*
	2837	3,059,410; Y	-*
	2907	3,063,077; 2R	1360{}6347*
	2967	19,180,631; 3R	close to *CG5555*, *CG14282*, *myd*
	3114	11,201,707; X	gypsy5{}103
pogo	166	30,374,902; 3R	close to *hdc*
	222	15,035,178; 3L	*Sytβ*
	235	21,531,991; 2R	*Egfr* *lncRNA:CR44725*
	941	15,069,400; X	*Lsd-2*
	1021	15,190,453; 2R	close to *igl*
	1202	21,254,529; X	*Mnr*
	1938	1,684,964; 3L	*CG7991*
	1972	14,688,291; 3R	*Meltrin*
	1993	22,113,023; 2L	NTs hotspot*
	2040	2,801,161; 3R	Pzl*
	2081	7,839,017; 2R	*Dgk*
	2139	10,889,337; 3L	close to *OXA1L*
	2166	17,730,485; 2L	*CadN*
	2280	18,913,439; 3R	close to *Sgsh*
	2410	20,456,532; 2L	*CG31687*
	2615	22,676,271; 3L	*Jhbp6*
	2768	17,503,189; 3L	*Oatp74D*
	3136	24,834,544; 3L	-*
S-element	67	9,950,745; 2R	*Mef2*
	415	23,313,720; 2L	-*
	940	1,664,262; 3R	Myo81F*
	1611	23,143,313; 3L	-*
	2290	6,076,695; 2L	-
	2353	3,827,600; 3R	-
	2555	5,674,700; 2R	Kune*
	2660	4,343,666; 2R	Gprk1*
	2730	25,200,700; 3L	CR40354*
	3019	20,099,984; X	3S18{}177
	3138	5,701,549; 2R	vlc*
	3528	23,400,788; 2L	-*
Tc1	2168	19,339,397; 3L	close to *CG32206*
Tc3	474	27,356,129; 3L	Dbp80*
	578	3,058,866; 3R	Pzl*
	3086	2,884,603; 3R	Pzl*
transib1	67	9,706,842; 2R	*CG1773*
	189	21,115,833; 3L	*ko*
	410	15,043,410; 2L	close to *Su(H)*
	718	26,170,465; 3L	CR41320*
	1760	2,221,072; 2L	Ir40a*
	1954, 1955	282,647; 3L	*RhoGEF3*
	1973	14,535,266; 3R	*Pde6* *lncRNA:CR46023*
	1974, 2041, 2042	5,533,505; 3L	close to *CG13285*
	2026	8,675,495; X	-
	2069	16,135,549; 2R	*spin*
	2137	3,473,173; 3L	*CG42324*
	2183	4,285,753; 3R	*cpx*
	2302	11,134,998; 3R	close to *mAcon2*
	2660	4,322,642; 2R	Gprk1*
	2877, 2878	15,028,387; 2L	*CG33310* *GABA-B-R1*
	2932	23,188,307; 2L	-*
	3138	5,787,158; 2R	-*
	3782	22,314,057; 3L	*Ten-m*
	3783	19,656,233; X	*Elys*
transib2	2375	958,213; 2R	-*
	2528, 2530, 2531	2,831,535; Y	-*
	2789	14,049,394; 3R	-
	3233	3,146,809; Y	-*
	3311	3,593,807; Y	ORY*
	3389	22,662,667; 2L	CG40006*

**Table 4:** Significant GO terms identified using FlyEnrichr and the lists of hit/close genes associated with A. Horezu_LaPeri specific (non-reference) NTs insertions; and B. Horezu_LaPeri specific and conserved (reference) NTs insertions.

GO Term	Genes	*p* Value
A. Horezu_LaPeri-specific NT insertions		
cellular metal ion homeostasis (GO:0006875)	*RhoGEF3*, *Pde6*, *Pde1c*, *CG8910*, *Glut1*, *GABA-B-R1*, *Dgk*, *cpx*, *CG34353*	1.34 × 10^−9^
temperature compensation of the circadian clock (GO:0010378)	*bru3*, *Pde6*, *Pka-C1*, *CG8910*, *CG32085*, *Pde1c*, *GABA-B-R1*, *Dgk*, *CG34353*	7.49 × 10^−8^
female mating behavior (GO:0060180)	*bru3*, *Pde6*, *Pka-C1*, *CG8910*, *Pde1c*, *Glut1*, *GABA-B-R1*, *mub*, *CG34353*	7.49 × 10^−8^
RNA–gene interaction with Putative Regulatory mir-4	*Mef2*, *bru3*, *Pka-C1*, *CG14459*, *Dbp80*, *CG7991*, *Dgk*, *cpx*, *CG42324*, *Nlg4*, *CG43373*, *CG8910*, *Pde1c*, *ORY*, *spin*, *GABA-B-R1*, *mam*, *mub*, *CG15221*	4.87 × 10^−7^
regulation of terminal button organization (GO:2000331)	*CG8910*, *CG14459*, *Pde1c*, *Glut1*, *CadN*, *GABA-B-R1*, *mub*, *CG34353*	1.00 × 10^−6^
regulation of muscle tissue development (GO:1001861)	*Mef2*, *Kank*, *Ten-m*, *CadN*, *beat-Ia*, *Egfr*, *Dys*	4.00 × 10^−6^
modulation of chemical synaptic transmission (GO:0050804)	*Pka-C1*, *cpx*, *Frq2*, *Nlg4*, *Dys*	7.00 × 10^6^
R7 cell differentiation (GO:0045466)	*Ten-m*, *CadN*, *rau*, *Egfr*	2.20 × 10^−5^
positive regulation of intracellular signal transduction (GO:1902533)	*Mef2*, *rau*, *mam*, *cpx*, *Egfr*	0.00014
3′,5′-cyclic-GMP phosphodiesterase activity (GO:0047555)	*Pde6*, *Pde1c*	0.000217
rhodopsin biosynthetic process (GO:0016063)	*Pka-C1*, *Gprk1*, *Dgk*, *Frq2*, *Egfr*, *AstA-R1*	0.000305
temperature response-defective phenotype	*CG8910*, *GABA-B-R1*, *mub*, *CG34353*	0.007
B. Horezu_LaPeri specific and conserved NT insertions		
response to alcohol (GO:0097305)	*bru3*, *Pka-C1*, *nAChRalpha4*, *Syt7*, *CG8910*, *Snap25*, *GABA-B-R1*, *CG18208*, *mub*, *CG34353*, *CG17684*, *MFS17*, *Ten-a*	3.15 × 10^−8^
sensory perception of touch (GO:0050975)	*AGO2*, *hiw*, *Usp2*, *Ank*	0.0001834
increased fecundity phenotype	*Pde1c*, *AGO2*, *Parp*, *kl-5*, *Egfr*, *Ank*	0.006329
radiation resistant phenotype	*rl*, *zfh2*, *sxc*	0.009606
circadian rhythm defective phenotype	*RhoGEF3*, *CadN*, *Egfr*, *Dys*, *Ank*	0.0342

## Data Availability

Whole Genome Shotgun project has been deposited at DDBJ/ENA/GenBank under the accession JANZWZ000000000. The version described in this paper is version JANZWZ020000000. KP element consensus sequence has been deposited at GenBank under the accession number OQ693612.

## Supplementary Materials

The following supporting information can be downloaded at: https://www.mdpi.com/article/10.3390/insects14060494/s1, Figure S1: PCR validation of P-element insertions in the Horezu_LaPeri genome; Table S1: sequences of primers used in PCR to validate the presence of P elements in the Horezu_LaPeri genome; Table S2: conserved DNA NTs insertions identified in the Horezu_LaPeri genome using Genome ARTIST_v2 and the reference genome of *D. melanogaster* r6.48; Table S3: the total number of P-element insertions having both ends (TIRs) and intact TSDs identified in the Horezu_LaPeri genome using GA_v2; Table S4: new insertions identified using LoRTE v1.2 in the nanopore reads of the Horezu_LaPeri genome.
