# Supplementary material for: The Landscape of the DNA Transposons in the Genome of the Horezu_LaPeri Strain of Drosophila melanogaster
Source: Insects. 2023 May 25;14(6):494. doi: 10.3390/insects14060494 (PMC10299278; doi:10.3390/insects14060494)
Supplement: Supplementary file 1 [file insects-14-00494-s001.zip › Suppl_Figure_S1.pdf]

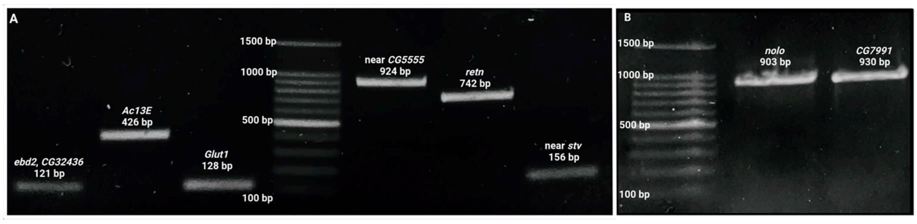

Supplementary Figure S1. PCR validation of P-element insertions in Horezu_LaPeri genome. **A)** Insertions detected with GA_v2 in *ebd2*, *CG32436*, *Ac13E*, *retn* and near *CG5555* and near *stv* genes. **B)** Insertions detected with GA_v2 in *nolo* and *CG7991* genes, amplified with double volume of MM11 primer. Position of the bands relative to the 100 bp DNA Ladder (Promega) and the estimated size of specific amplicons are represented. Edited with BioRender.com (accessed on 04 May 2023).
