# Supplementary material for: The Landscape of the DNA Transposons in the Genome of the Horezu_LaPeri Strain of Drosophila melanogaster
Source: Insects. 2023 May 25;14(6):494. doi: 10.3390/insects14060494 (PMC10299278; doi:10.3390/insects14060494)
Supplement: Supplementary file 1 [file insects-14-00494-s001.zip › Suppl_Table_S1.pdf]

Supplementary Table S1. Sequences of primers used in PCR to validate the presence of P elements in Horezu_LaPeri genome. The sequence of MM11 primer is: CGACGGGACCACCTTATGTTATTTC.

**Primer pairs**	**Genomic region**	**Sequence from 5' to 3'**	**Expected PCR product size (bp)**
P1 + MM11	*ebd2, CG32436*	AGGGATTGAGCATAGTGAACCG	121
P2 + MM11	*Glut1*	ATGCTTACTGACAGAGTGAGCG	128
P3 + MM11	close to *stv*	GGCGGTTTCAGACCTTAATTGG	156
P4 + MM11	*lncRNA:CR45233*	AACCGCTCAAACTTGTTTTCCA	295
P5 + MM11	*lncRNA:CR43626*	TGTTCATGTTTGATCTGCTGCC	379
P6 + MM11	*NFAT*	TAGACCGTAGTTCGTAAGTCGC	410
P7 + MM11	*Ac13E*	GACTGCCAATTGTGGAATACCC	426
P8 + MM11	*spri*	CAACATCCACTATGCACTCTGC	646
P9 + MM11	close to *CG43658*	ATCACTTTCAAAACGGTGCAGG	738
P10 + MM11	*retn*	GACCGACCCAAATGATTTCTGC	742
P11 + MM11	gipsy5{}103	GTCTAATTCTGGGTGGTAGGGC	797
P12 + MM11	*Cyp6g2*	CCGTTCTACGACTACACTCTCG	873
P13+ MM11	*CG7991*	TCAATTGAATGCCCTCAAAGCG	893
P14 + MM11	*nolo*	TGACAACATGGGTCTCCACC	903
P15 + MM11	close to *CG5555, CG31475*	TAAGACCTTTGGTCACCTCGAC	924
