# Supplementary material for: The Landscape of the DNA Transposons in the Genome of the Horezu_LaPeri Strain of Drosophila melanogaster
Source: Insects. 2023 May 25;14(6):494. doi: 10.3390/insects14060494 (PMC10299278; doi:10.3390/insects14060494)
Supplement: Supplementary file 1 [file insects-14-00494-s001.zip › Suppl_Table_S2.pdf]

Supplementary Table S2. Conserved DNA NTs insertions identified in the Horezu_LaPeri genome using Genome ARTIST_v2 and the reference genome of *D. melanogaster* r6.48. The annotated insertions are mentioned by their FlyBase identifier of the transposable element, while the unannotated insertions are mentioned by the coordinate where the NT is located. Insertions marked with an asterisk (*) stand for NTs located in heterochromatic regions.

NT name	Contig number	Insertion coordinate; host chromosome	Hit/Nearby Genes/NTs	FlyBase annotation
**Bari1**	595	Bari1{}Jheh^{Bari1855}; 2R	*asRNA:CR46485* and close to *Jheh2*	Yes
	1117, 2174, 2175, 2176, 2177, 2467, 2468, 2469	Bari1 cluster from h39 region; 2R: 180,603..287,454	-	No
	2099	Bari1 cluster; X	close to *28SrRNA:Ψ:CR45855**	No
	2280, 2281, 2966, 2967	Bari1{}Cyp12a4$^{Bari1-1386}$; 3R	close to *Cyp12a4, Cyp12a5* and *Ppcs*	Yes
	3003		Most probably in 2R	
	3469		Unresolvable	
**Bari2**	284, 1991	Bari2{}506; 2L	*nompB*	Yes
	331	Bari2{}1537; 4	*unc-13*	Yes
	711	22,570,232; X	-*	No
	808	4,804,322; 2R	*lncRNA:CR46477** NTs hotspot	No
**HB**	253	HB{}1517; 4	*zfh2* NTs hotspot	Yes
	294	HB{}3970; HB{}3969; 3L	-	Yes
	331	HB{}1548; 4	*PlexA* NTs hotspot	Yes
		HB{}1552; 4	*apolpp*	Yes
		HB{}1535; 4	NTs hotspot	Yes
	407, 2076, 2183	2918837; 2R	-*	No
	506, 2183	HB{}1194; 3R	*CG34357*	Yes
	511	HB{}761; 2R	-	Yes
	586	HB{}1219; 3R	*Cerk*	Yes
	616, 3208	HB{}1831; 3R	close to *Nlg3*	Yes
	646	24,251,060; 3L	*Snap25**	No
	711	HB{}276; X	*fog**, *CG42346**	Yes
		HB{}3716; X	*lncRNA:CR44997**	Yes
	773, 2613	HB{}1155; HB{}1151; 3L	NTs hotspot*	Yes
	808, 2940	HB{}5077; 2R	close to *conu** NTs hotspot	Yes
	1048	HB{}1242; 3R	*Fer1*	Yes
	1099	HB{}86; X	*CG15347*	Yes

	1140	HB{}3068; X	*hiw*	Yes
	1238	HB{}248; X	*Usp2**	Yes
	1371	HB{}134; X	*hiw, CG5541*	Yes
	1724	27,150,425; 3L	*vtd**	No
	1771	HB{}227; X	*DIP1**	Yes
	1989	HB{}482; 2L	close to *del*	Yes
	1991	HB{}512; 2L	*CG2225*	Yes
		HB{}519; 2L	-*	Yes
		HB{}2269; 2L	close to *eEF2*	Yes
		HB{}5495; 2L	close to *tsh**	Yes
	1993	HB{}529; 2L	*lncRNA:CR46480**<br>NTs hotspot	Yes
		HB{}524; 2L	NTs hotspot*	Yes
	2047	HB{}1183; 3R	*CR33294**	Yes
	2047, 2048	HB{}1185; 3R	*aux*	Yes
	2142	27,538,935; 3L	-*	No
	2322	HB{}1605; 3R	*heph*	Yes
	2555	HB{}674; 2R	*Ugt50B3**<br>NTs hotspot	Yes
	2658	4,461,263; 2R	*Gprk1**	No
	2661	4,200,622; 2R	-*	No
	2675	HB{}2180; 3L	close to *CG7133*	Yes
	2724	2,774,587; 2R	*CG41378**	No
	2831	26,229,932; 3L	close to *CG40160**	No
	2904	1,735,440; 2R	*CG41520**	No
	3024	3,467,608; 2R	close to *CR45227**	No
	3029		Unresolvable	
	3138	HB{}703; 2R	NTs hotspot*	Yes
	3500	HB{}887; 2R	*lov*	Yes
		HB{}892; 2R	close to *Ebp*	Yes
**hobo**	460	25,849,868; 3L	*l(3)80Fg**	No
	482, 512, 2183	H{}4523; 3R	-	Yes
	2024	22,465,193; 2R	*CG10384*	No
	2123	12,300,267; 3L	-	No
	2229	13,677,674; 2R	*tei*	No
	2279	H{}4815; 3R	-	Yes
	2654	943,071; 3R	*Myo81F**	No
	2719	H{}2157; X	-*	Yes
	2908	2,628,132; 3R	*Pzl**	No
	3135	24,775,148; 3L	-*	No
	3172	2,495,671; 2R	-*	No
	3451	12,090,664; X	*lncRNA:CR43960,*<br>*Ten-a,*<br>*lncRNA:CR32658*	No

hopper	11, 1981	694,545; Y	-*	No
	407, 2076	2,918,417; 2R	-*	No
	474, 2401	27,327,694; 3L	*Dbp80**	No
	564, 2163	2,311,266; 2R	-*	No
	744	2,906,148; 3R	*Pzl**	No
	800, 2507	1,638,912; 2R	-*	No
	874	hopper{}643; 2R	*sxc**<br>NTs hotspot	Yes
	892	hopper{}36; X	*lncRNA:CR44999*	Yes
	898, 2992	24,147,270; 3L	*Snap25**	No
	908	hopper{}1210; 3R	*atms, CG44098*	Yes
	910	hopper{}634; 2R	*Nipped-A**<br>NTs hotspot	Yes
	962	590,754; 2R	*DIP-λ**	No
	1011	hopper{}6535; 3R	*Myo81F**	Yes
	1178	hopper{}105; X	*CG44422*	Yes
	2155	hopper{}1031; 3L	NTs hotspot	Yes
	2308	2,050,704; 2R	*CG17684**	No
	2371	1,464,035; 2R	-*	No
	2374	1,263,557; 2R	*CG40191**	No
	2461	hopper{}6534; 3L	-*	Yes
	2613	hopper{}1181; 3L	*CG34031**<br>NTs hotspot	Yes
	2631	26,844,390; 3L	-*	No
	2908, 3375	2,668,075; 3R	*Pzl**	No
	3103		Unresolvable	
	3265	1,273,068; 3R	*Myo81F**	No
	3602	hopper{}4661; 2L	-	Yes
		hopper{}4688; 2L	-	Yes
	3769	hopper{}4663; 2L	-	Yes
mariner2	73, 1993	mariner2{}1651; 2L	*Marf1**<br>NTs hotspot	Yes
	329, 2723	2,793,469; 2R	*CG41378**	No
	332, 2114	mariner2{}1523; 4	close to *gw*	Yes
	460	25,845,385; 3L	*l(3)80Fg**	No
	583, 2011	mariner2{}1819; 3L	NTs hotspot*	Yes
	589, 2906, 2907, 3013, 3213, 3214	3,070,964; 2R	*dpr21**	No
	711	mariner2{}274; X	*lncRNA:CR44997**<br>NTs hotspot	Yes
		mariner2{}3718; X	-*	Yes
	759	2,652,263; 3R	*Pzl**	No
	808	mariner2{}5092; 2R	l*ncRNA:CR46477**	Yes
	1118, 1992	mariner2{}522; 2L	NTs hotspot*	Yes

	1153	mariner2{}198; X	-	Yes
	1155	mariner2{}225; X	-*	Yes
	1264	mariner2{}252; X	*CG17600**, INE-1{}2444	Yes
	1611	mariner2{}3947; 3L	close to *CG33217**	Yes
	1991	mariner2{}513; 2L	*nolo*	Yes
	2011	mariner2{}1821; 3L	*AGO3**	Yes
	2076	2,948,288; 2R	*Sep1*	No
	2114	mariner2{}1522; 4	*Ekar*<br>NTs hotspot	Yes
	2142	27,856,259; 3L	*eIF4B**,<br>*lncRNA:CR46482**	No
	2287	mariner2{}628; 2R	*Nipped-A**	Yes
	2298	3,469,757; 3R	*Parp**	No
	2308	2,109,132; 2R	*CG17684**	No
	2613	mariner2{}1130; 3L	NTs hotspot*	Yes
	2614	mariner2{}1115; 3L	NTs hotspot*	Yes
	2616	mariner2{}767; 2R	*Glo1*<br>NTs hotspot	Yes
	2658, 2659, 2739	4,425,204; 2R	*Gprk1**	No
	2664	3,931,953; 2R	*uex**	No
	2724	2,678,501; 2R	-*	No
	2752, 3472	3,287,197; 2R	-*	No
	2904	1,777,959; 2R	*CG41520**	No
	2908	2,671,981; 3R	*Pzl**	No
	3368	558,088; 3R	-*	No
	3565	23,042,079; X	*CG12061**	No
**NOF**	578	3,123,108; 3R	*Pzl**	No
	2394	NOF{}1401; 3R	-	Yes
**pogo**	817, 990		Unresolvable	
	1962	pogo{}895; 3L	-	Yes
	1990	pogo{}4759; 2L	-	Yes
	2637		Most probably in X	
	2719, 3249, 3445		Unresolvable	
	3602	pogo{}433; 2L	-	Yes
	3835	pogo{}6553; 3L	-*	Yes
**S-element**	202, 2401	27,387,339; 3L	*Dbp80**	No
	202	27,395,632; 3L	*Dbp80**	No
	219, 2353	3,750,087; 3R	close to *CG41128**	No
	219	3,749,248; 3R	close to 1360{}6424*	No
	222	S{}AGO2[S1008]; 3L	*AGO2*	Yes
	253, 2454	S{}1513; 4	-	Yes
	286	25,610,028; 3L	*lovit**	No

455	25,738,461; 3L	close to 17.6{}6492*	No
455, 2952	25,733,788; 3L	-*	No
469, 2990, 2991	27,281,198; 3L	*Dbp80**	No
511	S{}758; 2R	*Adf1*	Yes
529, 2310	S{}746; 2R	-	Yes
586	S{}1217; 3R	*Tim17a2, Hph*	Yes
646	24,325,461; 3L	close to *nvd**	No
711	S{}260; X	-*	Yes
744	2,682,905; 3R	*Pzl**	No
773		Unresolvable	
871	852,569; 3R	*Myo81F**	No
	861,086; 3R	*Myo81F**	No
874	S{}652; 2R	-*	Yes
881	S{}3280; 2R	-*	Yes
908	S{}1207; 3R	*smash*	Yes
	S{}1208; 3R	-	Yes
941	S{}135; X	-	Yes
1011	1,343,654; 3R	*Myo81F**	No
	1,300,818; 3R	*Myo81F**	No
1153	S{}195; X	*CG15446*<br>*stg1*	Yes
1264	S{}1647; X	-*	Yes
	S{}3184; X	-*	Yes
1602	S{}1026; 3L	*NUCB1*	Yes
1616		Unresolvable	
1933	1,526,163; 2R	*Maf1**	No
	1,529,938; 2R	-*	No
1960		Unresolvable	
1968, 1985	S{}1347; 3R	-	Yes
	S{}4761; 3R	-	Yes
1991	S{}4784; 2L	Max{}4867	Yes
1993	S{}532; 2L	*CG42748**	Yes
	S{}533; 2L	*CG42748**	Yes
2011	S{}5868; 3L	*AGO3**<br>NTs hotspot	Yes
2019, 2483	S{}1448; 3R	*CG14518*	Yes
2052	1,642,861; 3R	*Myo81F**	No
	1,694,280; 3R	*Myo81F**	No
2116	2,421,564; 3R	*Myo81F**	No
2155	S{}1035; 3L	-	Yes
2172	S{}1237; 3R	-	Yes
2173	322,894; 2R	close to *DIP-λ**	No
2183	S{}1196; 3R	*CG34357*<br>NTs hotspot	Yes

		S{}3902; 3R	-	Yes
	2225	693,800; 2R	*DIP-λ**	No
	2252		Unresolvable	
	2290	S{}312; 2L	*stai*	Yes
	2308	2,092,186; 2R	*CG17684**	No
	2353	3,792,255; 3R	-*	No
	2430	2,195,782; 2R	*CG17684**	No
	2454	S{}1511; 4	NTs hotspot	Yes
	2490, 3534, 3535	23,375,679; X	-*	No
	2494		Unresolvable	
	2507	1,590,098; 2R	close to *CG17691**	No
	2537		Unresolvable	
	2571	23,351,597; X	-*	No
	2579		Most probably in Y	
	2596	25,414,450; 3L	*lovit**	No
	2613	S{}1150; 3L	-*	Yes
		S{}1156; 3L	-*	Yes
		S{}1121; 3L	*CkIIalpha**<br>NTs hotspot	Yes
	2654	942,968; 3R	*Myo81F**	No
	2658	4,468,942; 2R	*Gprk1**	No
		4,461,891; 2R	*Gprk1**	No
	2692		Unresolvable	
	2789	S{}1355; 3R	-	Yes
	2812	4,057,226; 3R	-*	No
	2873	1,155,538; 3R	*Myo81F**	No
	2908, 2909	2,684,623; 3R	*Pzl**	No
	2915, 2944		Unresolvable	
	3029	S{}6581; Y	-*	Yes
	3164, 3168		Unresolvable	
	3243	27,086,583; 3L	close to *CG40228**	No
	3293	31,671; Y	*kl-5**	No
	3306	S{}665; 2R	*stw**<br>NTs hotspot	Yes
	3347	23,915,527; 3L	*CG40470**	No
	3368	611,450; 3R	*Myo81F**	No
	3395		Unresolvable	
	3396, 3534	23,355,679; X	-*	No
	3602	S{}447; 2L	NTs hotspot	Yes
	3703		Most probably in 2R	
**S2**	758		Unresolvable	
	871	833,034; 3R	*Myo81F**	No
	1380	S2{}249; X	close to<br>invader1{}3257,	Yes

			*CG14613**, *CG12576**, *Usp2**	
	1989	S2{}491; 2L	close to G5{}492, *nrv3*<br>NTs hotspot	Yes
	2076	2,961,468; 2R	*Scp1**	No
	2116	2,476,278; 3R	*Myo81F**	No
	2183	S2{}1200; 3R	NTs hotspot	Yes
	2264		Unresolvable	
	2280	S2{}1385; 3R	*Xrp1*	Yes
	2308	2,054,988; 2R	*CG17684**	Yes
	2553	S2{}1710; 2R	*Ir41a**<br>NTs hotspot	Yes
	2613	S2{}1120; 3L	*CkIIalpha**<br>close to S{}1121, *RpL10**	Yes
		S2{}1172; 3L	*nAChRα4**<br>NTs hotspot	Yes
	2664	3,904,119; 2R	*uex**<br>close to 1360{}6353	No
	2812	4,077,592; 3R	close to *CG42402**	No
	2832	S2{}6583; 3L	-*	Yes
		S2{}6582; 3L	*l(3)80Fg**	Yes
	2875, 2876	1,065,890; 3R	*Myo81F**	Yes
**Tc1**	11, 1981	691,853; Y	-*	No
	185		Unresolvable	
	219, 2353	3,746,649; 3R	1360{}6424*	No
	263	Tc1{}617; 2R	NTs hotspot*	Yes
	289, 2661	4,165,977; 2R	-*	No
	331	Tc1{}1544; 4	-	Yes
		Tc1{}1549; 4	*PlexA*	Yes
		Tc1{}1550; 4	*CamKII*<br>NTs hotspot	Yes
	331, 2306	Tc1{}1556; 4	*Kif3C*	Yes
		Tc1{}1557; 4	*Kif3C*	Yes
	333	Tc1{}1211; 3R	close to *CG14658* and *CG14659*	Yes
	521, 539, 1883, 2372	Tc1{}6593; 2R	*Yeti**	Yes
	521, 539, 2372	Tc1{}6594; 2R	-*	Yes
	539, 2372	1,284,699; 2R	-*	No
	586	Tc1{}1216; 3R	*dpr16*	Yes
	711	Tc1{}4352; X	NTs hotspot*	Yes
	714, 715	24,072,383; 3L	close to *Snap25**	No
	714	24,033,292; 3L	*CG40045**	No
	756	26,816,906; 3L	-*	No
	790, 2887, 3137	24,922,073; 3L	-*	No

	798	188,717; Y	*PRY**	No
	874	Tc1{}641; 2R	close to *sxc**<br>NTs hotspot	Yes
	993	3,023,693; 3R	*Pzl**	No
	1246	Tc1{}4353; X	close to *Coa7**	Yes
	1464	Tc1{}172; X	close to *Cdc42*	Yes
	1883	1,285,426; 2R	-*	No
	2111	Tc1{}826; 2R	close to *RpS23* and *CG8468*	Yes
	2114	Tc1{}1529; 4	*bt*	Yes
		Tc1{}1531; 4	*MED26*<br>NTs hotspot	Yes
		Tc1{}1633; 4	*CG11360*<br>NTs hotspot	Yes
	2166	Tc1{}403; 2L	close to *CG44476* and *CG15152*	Yes
	2372	1,076,978; 2R	*rl**	No
	2455	Tc1{}1485; 4	*Ank*<br>NTs hotspot	Yes
		Tc1{}1500;4	*Syt7*	Yes
	2459	Tc1{}6596; 3L	-*	Yes
		26,566,093; 3L	-*	No
	2478		Unresolvable	
	2599, 2932, 2933, 2985	23,178,990; 2L	-*	No
	2830, 2831, 3471	Tc1{}6595; 3L	*CG41284**	Yes
	2905	3,130,514; 2R	*dpr21**	No
	2942	Tc1{}5005; 2R	*Nipped-B**<br>NTs hotspot	Yes
	2986		Unresolvable	
	2994	Tc1{}5901; 3L	*AGO3**<br>NTs hotspot	Yes
	3011	Tc1{}1080; 3L	close to *CG5664*	Yes
	3306	Tc1{}663; 2R	close to *stw**	Yes
	3565	Tc1{}6597; X	*CG12061**	Yes
**Tc3**	333	Tc3{}5089; 3R	*Cdep*	Yes
	482, 512	Tc3{}5024; 3R	-	Yes
	714	24,088,971; 3L	*Snap25**	No
	1494	502,195; Y	*kl-3**	No
	1993	Tc3{}2088; 2L	-*	Yes
	2183	Tc3{}3768; 3R	-	Yes
	2264	22,749,005; 2L	*CG40006**	No
	2308	2,048,629; 2R	*CG17684**	No
	2942	Tc3{}5138; 2R	*Nipped-B**	No
**transib1**	402	transib1{}4274; 2R	NTs hotspot*	Yes
	529, 740, 2310	6,481,813; 2R	NTs hotspot	No

	578	3,144,932; 3R	*Pzl**	No
	611, 2905	3,140,380; 2R	*dpr21**	No
	646	24,281,995; 3L	*Snap25**	No
	855	494,613; Y	*kl-3**	No
	2005	450,257; 3R	-*	No
	2479	Unresolvable		
	2658	transib1{}4274; 2R	-*	Yes
	2873	1,208,948; 3R	*Myo81F**	No
	2933	Unresolvable		
	3135	24,739,452; 3L	*scro**	No
	3175	27,128,137; 3L	-*	No
	3497	23,511,907; 2L	-*	No
**transib2**	164, 236, 2444, 2445	transib2{}2891; 3L	NTs hotspot*	Yes
	202, 2733, 3524	27,466,293; 3L	-*	No
		27,583,897; 3L	-*	No
	221, 2061	transib2{}1003; 2R	-	Yes
	329, 2724	2,782,741; 2R	*CG41378**	No
	504	1,964,060; 3R	*Myo81F**	No
	529, 740, 2310	transib2{}754; 2R	NTs hotspot	Yes
	601, 2884	transib2{}6602; 3L	-*	Yes
	756, 2461, 2462	26,667,964; 3L	-*	No
	808	transib2{}5064; 2R	NTs hotspot*	Yes
	914	Most probably in Y		
	1117, 1125	68,418; 2R	-*	No
	1117	73,644; 2R	-*	No
		79,257; 2R	-*	No
	1158	transib2{}157; X	*Bx*	Yes
	1159	23,286,743; X	-*	No
	1264	transib2{}259; X	NTs hotspot*	Yes
	1399	541,878; Y	*kl-3**	No
	1993	transib2{}1578; 2L	NTs hotspot*	Yes
	2096	633,789; Y	*kl-2**	No
	2142, 2144	27,636,857; 3L	-*	No
	2173	346,515; 2R	*DIP-λ**	No
	2308, 2430	1,877,936; 2R	*CG17684**	No
	2478	Unresolvable		
	2524	2,159,461; 3R	*Myo81F**	No
	2596	2,544,661; 3L	*lovit**	No
	2609	transib2{}6603; Y	-*	Yes
	2658	transib2{}3740; 2R	close to *Rpl38**	Yes
	2830	26,380,341; 3L	-*	No
	2887	transib2{}6601; 3L	-*	Yes
	2942, 3631	transib2{}5058; 2R	*Nipped-B**	Yes

			NTs hotspot	
	3002	Unresolvable		
	3193	23,008,563; 2L	*Cht10**	No
	3306	transib2{}671; 2R	*CG30440**	Yes
	3387	360,528; Y	*kl-3**	No
	3501	1,553,097; Y	-*	No
	3602	transib2{}1688; 2L	NTs hotspot	Yes
	3753	Most probably in Y		
**transib3**	93, 2266, 3389, 3390	22,667,207; 2L	*CG40006**	No
	164, 2445	transib3{}2951; 3L	invader2{}2886, Doc{}2376*	Yes
	512, 2183	transib3{}1202; 3R	-	Yes
	578	3,119,987; 3R	*Pzl**	No
	585	2,275,172; 3R	*Myo81F**	No
	673	2,609,128; 3R	*Pzl**	No
	771	transib3{}4416; X	-*	Yes
	904, 1017	transib3{}2950; 3L	-*	Yes
	1017	transib3{}2949; 3L	*AGO3**	Yes
	1993	transib3{}567; 2L	*Marf1**, invader5{}1644	Yes
	2009, 3172	2,519,371; 2R	*MFS17**	No
	2264, 2265, 3391	22,684,559; 2L	*CG40006**	No
	2293	1,835,441; 3R	*Myo81F**	No
	2429	584,669; Y	*kl-2**	No
	2455	transib3{}1705; 4	*Syt7* NTs hotspot	Yes
	2615, 3212	transib3{}1112; 3L	-*	Yes
	2873	1,144,915; 3R	*Myo81F**	Yes
	2908	2,612,008; 3R	*Myo81F**	Yes
	3161	3,291,506; 3R	-*	Yes
	3306	transib3{}670; 2R	*CG30440**	Yes
	3755	3,309,826; 3R	-*	No
	3758	3,296,524; 3R	-*	No
**transib4**	996	transib4{}75; X	-	Yes
	1264	transib4{}251; X	-*	Yes
	1993	transib4{}1579; 2L	diver2{}2295*	Yes
	2052	1,528,843; 3R	*Myo81F**	No
	2189	Unresolvable		
	2287	transib4{}623; 2R	-*	Yes
	2293	Unresolvable		
	2658	transib4{}3834; 2R	*Stlk**	Yes
		transib4{}3815; 2R	-*	Yes
	3375	2,880,264; 3R	*Pzl**	No
	3692	Most probably in X		
