# Supplementary material for: The Landscape of the DNA Transposons in the Genome of the Horezu_LaPeri Strain of Drosophila melanogaster
Source: Insects. 2023 May 25;14(6):494. doi: 10.3390/insects14060494 (PMC10299278; doi:10.3390/insects14060494)
Supplement: Supplementary file 1 [file insects-14-00494-s001.zip › Suppl_Table_S3.pdf]

Supplementary Table S3. The total number of P-element insertions having both ends (TIRs) and intact TSDs identified in the Horezu_LaPeri genome using GA_v2.

Assembly_contig ID	Chromosome	Genomic coordinate	Hit/Nearby Genes/NTs	NT Orientation	TSD
Canu_491	3L	21,347,168	*ebd2, CG32436* intronic	+	GTTCCGCT
Canu_869	X	10,547,695	*pri* intronic	+	GGAGAGCC
Canu_1601	3L	3,250,477	*lncRNA: CR43626*	-	GCTCAAAT
Canu_1937	3L	959,438	*Glut1* intronic	+	CTTGGAAC
Canu_1938	3L	1,688,826	*CG7991* exonic	-	CGCCGTCC
Canu_2082	2L	21,697,891	*nolo* intronic	+	ACTCGGGG
Canu_2967	3R	19,180,631	close to *CG5555, CG31475*	-	GTTTGGAT
Canu_3114	X	11,201,707	gipsy5{}103	-	CGCCAGTG
CanuAlt_3182	3L	14,410,404	*lncRNA: CR45233*	+	GTCTGGCA
CanuAlt_3419	2R	12,188,531	*Cyp6g2* exonic	-	GTCTAGAC
FlyeAlt_1148	2R	23,635,173	*retn* intronic	-	GTCCATGG
FlyeAlt_1188	X	15,552,365	*Ac13E* intronic	+	GTATGAGC
FlyeAlt_1736	3L	13,477,235	close to *stv*	-	GTCTGATG
FlyeAlt_1795	X	17,562,651	close to *CG43658*	-	CGCCAGCT
FlyeAlt_2153	X	13,641,922	*NFAT* intronic	-	GGCCACGC
