# Supplementary material for: The Landscape of the DNA Transposons in the Genome of the Horezu_LaPeri Strain of Drosophila melanogaster
Source: Insects. 2023 May 25;14(6):494. doi: 10.3390/insects14060494 (PMC10299278; doi:10.3390/insects14060494)
Supplement: Supplementary file 1 [file insects-14-00494-s001.zip › Suppl_Table_S4.pdf]

Supplementary Table S4. New insertions identified using LoRTE v1.2 in nanopore reads of Horezu_LaPeri genome.

RepBaseID#Type/Family	Coverage	Genomic coordinates	Genomic region	Percentage of covered NT	Percentage of polymorph	Number of deletion
DM176_LTR#LTR/Gypsy	10	2R:7,606,220.. 7,606,623	*CG1358* intron	99.95	2.93	568
M4DM#DNA/CMC-Transib	13	2L:20,969,629..20,970,029	*CG42238* intron			
TRANSIB2#DNA/CMC-Transib	7	3R:14,049,171..14,049,575	noncoding region			
LINEJ1_DM#LINE/Jockey	1	2L:15,257,737..15,258,141	*yuri* exon			
LINEJ1_DM#LINE/Jockey	1	3R:22,199,769..22,200,183	*SKIP* intron and *lncRNA*:*CR45224*			
M4DM#DNA/CMC-Transib	4	X:18,356,645..18,357,047	INE-1{}4916			
Jockey-4_DAn#LINE/Jockey	1	3R:31,536,097..31,536,508	noncoding region			
